# Supplementary material for: Rab44 Deficiency Induces Impaired Immune Responses to Nickel Allergy
Source: Int J Mol Sci. 2023 Jan 4;24(2):994. doi: 10.3390/ijms24020994 (PMC9866195; doi:10.3390/ijms24020994)
Supplement: Supplementary file 1 [file ijms-24-00994-s001.zip › ijms-2110040-supplementary.pdf]

Supporting Information for

Rab44 deficiency induces impaired immune responses to nickel allergy

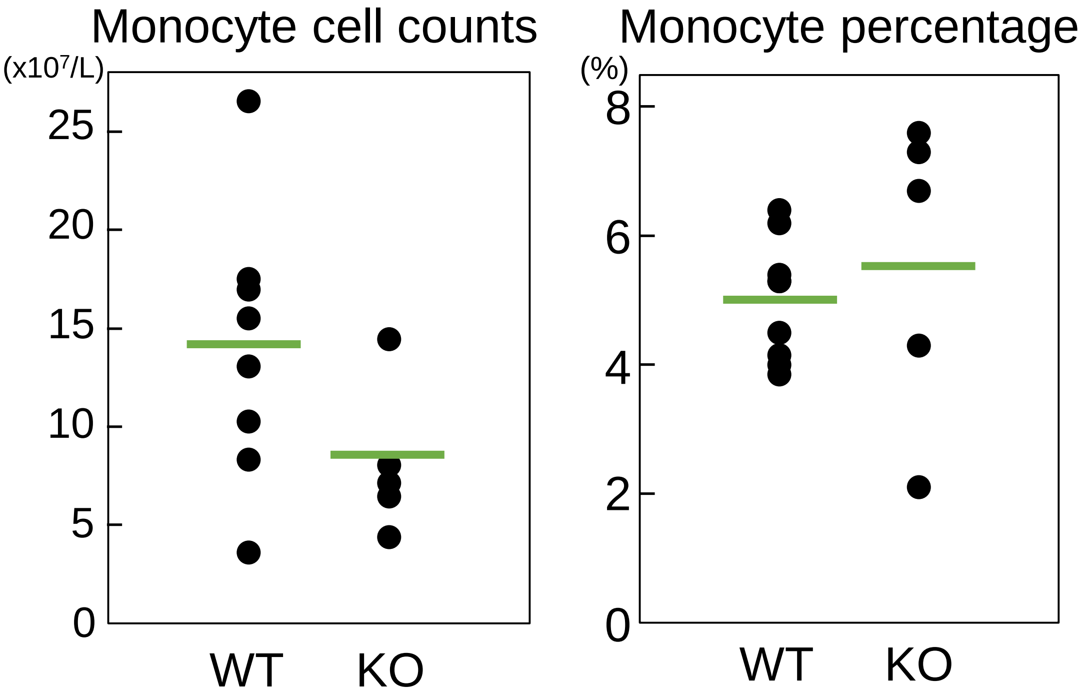

Figure S1. Different immune responses between wild-type (WT) and Rab44-knockout mice (KO) to LPS administration.
WT and Rab44-KO female mice were intraperitoneally injected with 0.2 mg of LPS (O55:B5). After 48 h of injection, the blood was analyzed with a haematology counter. Monocyte counts (left panel) and percentages in white blood cells (right panel).

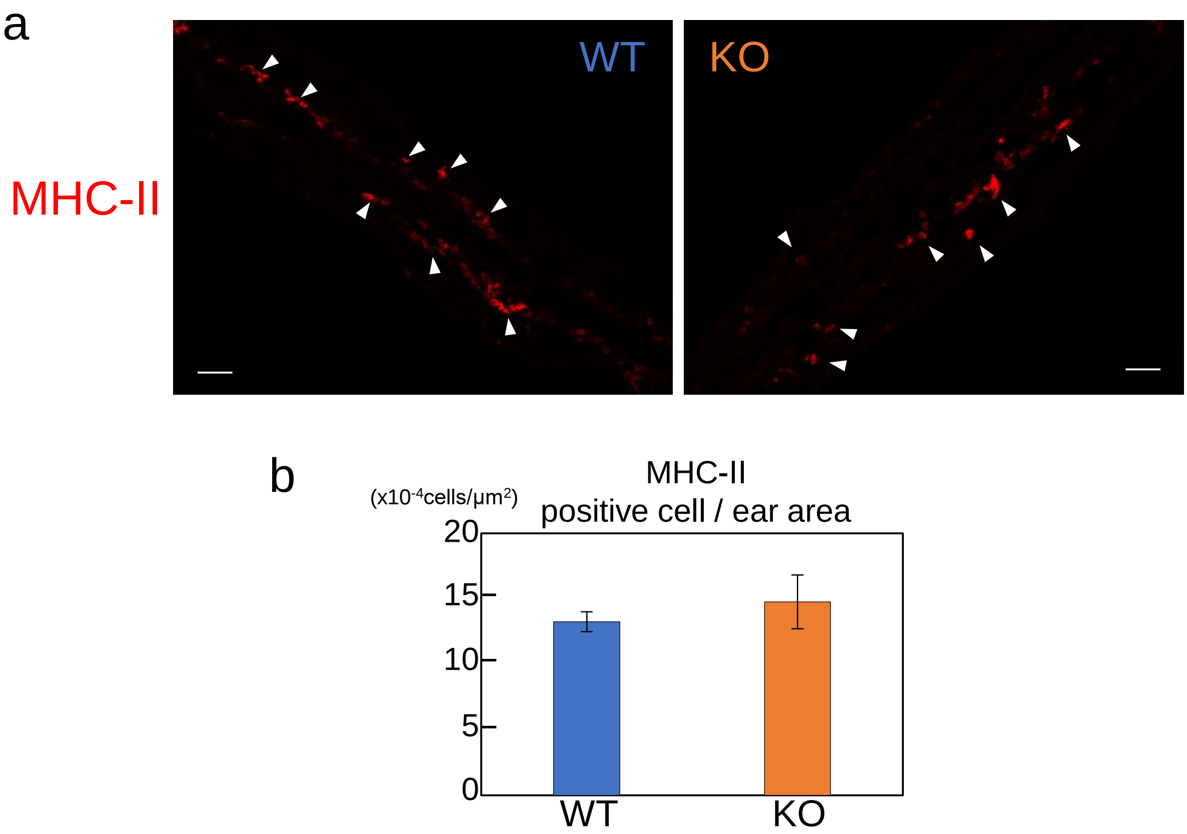

Figure S2. Immunofluorescence analysis of MHC-II positive cells in nickel - induced ear in wild-type (WT) and Rab44-knockout (KO) mice
(**a**) The fixed sections of the nickel-treated ear were blocked with 1.0% bovine serum albumin in PBS. The samples were incubated with mouse anti-MHC-II IgG (1:250), followed by fluorescent labelling with Alexa Fluor Alexa fluor 555-conjugated anti-mouse IgG. Bars: 50 μm. White arrowheads indicate the antibody reactive cells. (**b**) Quantitative analysis of MHC-II positive cells per μm^2 after visualization by microscopy. Data are represented as the mean ± S.E. of values from three independent experiments.
